# Brachial-Ankle Pulse Wave Velocity Mediates the Association between Increased Age and Risk of Sarcopenia among Chinese Patients with Type 2 Diabetes Mellitus

**DOI:** 10.1155/2023/3786342

**Published:** 2023-01-06

**Authors:** Jiahong Jiang, Ping Zhang, Zijun Zhou, Zeru Xu, Xiang Hu, Weihui Yu, Feifei Jiang, Yang Pan, Fei Wang, Jing Hong, Tingting Ye, Ke Xu, Hong Zhu

**Affiliations:** ^1^Department of Endocrinology, The First Affiliated Hospital of Wenzhou Medical University, Wenzhou, Zhejiang 325000, China; ^2^The Second People's Hospital of Xining, Xining, Qinghai 816000, China; ^3^Wenzhou Medical University, Wenzhou, Zhejiang 325000, China; ^4^Wenzhou Key Laboratory of Diabetes Research, Wenzhou, Zhejiang 325000, China

## Abstract

**Background and Aims:**

Arterial stiffness and sarcopenia are commonly seen in patients with type 2 diabetes mellitus (T2DM), and both are age-related diseases. However, few studies have addressed the causal relationship between age, arterial stiffness, and sarcopenia, especially in patients with T2DM. This study is aimed at investigating the relationship among age, arterial stiffness, and sarcopenia in patients with T2DM.

**Methods and Results:**

This cross-sectional study enrolled 557 inpatients with diabetes at the First Affiliated Hospital of Wenzhou Medical University, China, between June 2020 and July 2021. Patients who were diagnosed with T2DM and underwent examination of dual-energy X-ray absorptiometry, handgrip strength, 6-meter walk speed, and brachial-ankle pulse wave velocity (baPWV, a recognized indicator of arterial stiffness) were enrolled. A total of 447 patients were included. A dose-dependent relationship was found between age and sarcopenia. We also found a dose-dependent relationship between age and baPWV. Similarly, significant dose-dependent relationships were found across baPWV tertiles with higher prevalence of sarcopenia. Then, a mediation analysis was performed to explore the mediation effect of arterial stiffness on age-associated sarcopenia. We found that the prevalence of sarcopenia increased by 0.0115 (95% CI, 0.0028–0.0239) per 1 year increase in age by the mediation effect of baPWV and that the direct effect of aging on sarcopenia was 0.0441 (95% CI, 0.0101–0.0909) per 1 year older. baPWV mediated 20.5% of the positive relationship between increased age and the prevalence of sarcopenia.

**Conclusions:**

Elevated baPWV partially mediates the association of age and sarcopenia among patients with T2DM.

## 1. Introduction

Sarcopenia has been recognized as an age-related disease [1]. Some longitudinal studies have shown that the incidence of sarcopenia increases with age [2, 3]. In a prospective study among European men with a mean follow-up of 4.3 years, appendicular lean mass was found to have decreased significantly after the age of 50 years, while gait speed and grip strength showed a significant decrease after the age of 70 [2]. The Baltimore Longitudinal Study of Aging and the Health ABC study have also suggested that the rate of decline in leg muscle mass became steeper in older age, which was accompanied with a more substantial change in muscle strength [3].

In the vascular aging process, arterial stiffness occupies a central position [4]. Recently, a large-scale observational study on 80,415 healthy subjects from China has shown an age-related pattern of arterial stiffness [5]. A prospective cohort study has also found a positive association between age and arterial stiffness status [6]. Arterial stiffness and muscle loss are both age-related diseases [7]. The relationship between arterial stiffness and sarcopenia aroused our interest. For example, in a study of 1,518 healthy individuals aged 55 years or above, both sarcopenic indices of thigh muscle cross-sectional area and skeletal muscle mass were modestly but significantly associated with arterial stiffness in both male and female subjects [8].

According to the present studies, whether arterial stiffness plays a role in the relationship between age and sarcopenia among Chinese population has not been reported yet. A high prevalence of sarcopenia has consistently been reported among patients with type 2 diabetes mellitus (T2DM), compared with those with normoglycemic status [9]. Moreover, increased arterial stiffness is associated with impaired glucose metabolism and T2DM [10]. Diabetes-related arterial stiffness predicts all-cause and cardiovascular mortality in T2DM patients [11]. To evaluate arterial stiffness, pulse wave velocity (PWV) is a noninvasive approach, which is a more convenient and inexpensive technique and is widely used, compared with carotid-femoral pulse wave velocity (cfPWV) [5].

Thus, this study is aimed at examining the cross-sectional association of increased age and sarcopenia among Chinese patients with T2DM and to assess the mediation effect of increased arterial stiffness on this relationship and to investigate the effects of metabolic, nutritional, and cardiovascular indicators.

## 2. Material and Methods

### 2.1. Study Design and Participants

A total of 557 patients hospitalized at the Department of Endocrinology, the First Affiliated Hospital of Wenzhou Medical University, were enrolled in this cross-sectional study between June 2020 and July 2021.

In this study, we included 485 participants who fulfilled the following criteria: (1) they received the diagnosis of T2DM based on the American Diabetes Association's criteria [12], and (2) they consented to participate in dual-energy X-ray absorptiometry examination. Patients were excluded if they met the following criteria: (1) incomplete information on baPWV; (2) bilateral ankle-brachial index (ABI) < 0.9 (the measurement of baPWV can be biased in severe atherosclerosis of the lower extremity [13]); (3) acute stroke, acute myocardial infarction, hepatic failure, estimated glomerular filtration rate (eGFR) < 15 ml/min/1.73 m^2^, or terminal disease (including terminal cancer) with life expectancy less than 1 year; (4) severe arthritis; (5) chronically bedridden patients; and (6) unable to communicate with interviewers. The final sample was consisted of 447 participants. The protocol was approved by the Ethics Committee of the First Affiliated Hospital of Wenzhou Medical University. Written informed consent was obtained from each participant.

### 2.2. Sarcopenia Evaluation

To evaluate the risk of sarcopenia, we applied the SARC-CalF scale, which has been described in detail elsewhere [14]. A score of SARC − CalF ≥ 11 indicates a positive result for screening sarcopenia [15].

Sarcopenia was diagnosed in accordance with the Asian Working Group for Sarcopenia (AWGS) 2019 consensus [16] as follows: (1) low muscle mass measured by dual-energy X-ray absorptiometry (Prodigy Primo-81013GA series; software 11.40.004, GE Healthcare USA, China), SMI < 7.0 kg/m^2^ in men and <5.4 kg/m^2^ in women and (2) low handgrip strength as measured with a grip dynamometer (Camry, model: TH-01, China); the higher value was used after repeating the measurement twice, and the cutoff handgrip strength value was 26 kg for men and 18 kg for women; or (3) low physical performance defined as the mean value of 6 − meter walk speed < 1.0 m/s, repeated twice [16].

### 2.3. Measurement of baPWV

ABI and baPWV were measured with a networked arterial stiffness detection device (BP-203 RPE III, Omron Health Medical, China), as detailed elsewhere [5, 17]. The higher value of the left and the right sides of baPWV was used for the analysis. If an ABI < 0.9, the value of baPWV of the opposite side was used for analysis [5].

In this study, the patients were categorized into three groups according to baPWV, as follows: normal arterial stiffness (baPWV < 1400 cm/s) group, borderline arterial stiffness (1400 ≤ baPWV < 1800 cm/s) group, and elevated arterial stiffness (baPWV ≥ 1800 cm/s) group [18, 19].

### 2.4. Assessment of Age and Other Covariates

Information about age was collected from the electronic medical records, and the patients were divided into three tertiles (≤53 years, 54–65 years, and ≥66 years).

Other clinical parameters including anthropometric indicators, laboratory indicators, and lifestyle information were also collected. Height and weight were performed by trained nurse. Body mass index (BMI, low, <24 kg/m^2^; high, ≥24 kg/m^2^ [20]) was calculated as weight (kilogram)/height^2^ (meter^2^). Systolic blood pressure (SBP) was read on an automatic sphygmomanometer (HEM-7133, Omron Health Medical, China). Blood samples were drawn using standardized procedures. Alanine transaminase (ALT), creatinine, albumin, low-density lipoprotein cholesterol (LDL-c), vitamin D, hemoglobin A1c (HbA1c, < 7 or ≥ 7%), fasting blood glucose (FBG), and two-hour postload plasma glucose (2hPG) were measured by AutoAnalyzer (Beckman-Coulter AU 5800, US). FBG and 2hPG were collected from the steamed bread meal test. Vibration perception threshold (VPT, low, < 15 V; high, ≥ 15 V) was measured by a technician for easily assessing the risk of nephropathy [21], with a neurothesiometer (Semsiometer A200, China). Duration of diabetes (≤ 10 or > 10 years), smoking status (ever or never), and drinking status (ever or never) were also collected. Ever smokers (drinkers) included current and former smokers (drinkers). Current smoker was defined in detail elsewhere [19]. Current drinker was defined as having alcohol drinking past 30 days [22].

### 2.5. Statistical Analysis

Statistical analysis was performed using SPSS statistics V26.0. Two-sided *P* value < 0.05 was considered statistically significant. The characteristics of the included patients were described as mean ± SD, median (interquartile range), or number (percentages). One-way ANOVA for continuous variables and *χ*^2^ test for categorical variables were used to compare the basic characteristics of patients with sarcopenia and those without.

Multivariate logistic regression models were applied to estimate the odds ratio (OR) with 95% confidence interval (CI) for the risk of sarcopenia associated with increased age and baPWV among T2DM patients, with the normal arterial stiffness and low age groups as the reference categories; model 1 was adjusted for sex, smoking status, and drinking status. In model 2, we adjusted for model 1 plus SBP and VPT classes. Model 3 was then adjusted for model 2 plus BMI classes, ALT, creatinine, albumin, LDL-c, vitamin D, HbA1c classes, and diabetes duration classes. We standardized baPWV to have a mean of 0 and an SD of 1, because the ranges between baPWV and age tertiles were dramatically different.

Generalized linear regression models were performed to assess the association between age tertiles and baPWV, also adjusted for different models.

### 2.6. Mediation Analysis

Given the association between age, baPWV, and sarcopenia, a mediation analysis was performed by *Introduction to Mediation, Moderation, and Conditional Process Analysis* (version 4.1) to explore how the mediator (standardized baPWV) affects the relationship between the independent variable (age) and the outcome variable (sarcopenia). The mediation analysis was adjusted for model 3. Because the total effect model was not available with binary outcome variable (sarcopenia, yes or no), in the process, we further analyzed the total effect using a logistic regression model. The number of bootstrap samples was set at 5000. If the 95% CI of the effects did not include the null value, the effects were interpreted as statistically significant.

## 3. Results

### 3.1. Characteristics of the Study Population

The basic characteristics are presented in Table 1. Among the included patients, the mean (±SD) age was 58.2 ± 13.2 years, and 65.3% were men. Out of 447 patients, 90 were diagnosed with sarcopenia. In the final analyses, only two patients in the sarcopenia group were diagnosed as prostate cancer (2.22%), and both were postsurgical patients undergoing pharmacological treatment. Only one patient in the nonsarcopenia group was diagnosed as lung cancer (0.28%) and had undergone surgery and completed postoperative adjuvant chemotherapy. Patients with sarcopenia were older than those without sarcopenia, and they had higher SARC-CalF scores, baPWV, blood glucose level, diabetes duration, and VPT but lower ALT level and poorer nutrition status (including albumin, vitamin D, and BMI).

### 3.2. Association between Increased Age and Sarcopenia

As presented in Table 2, a positive relationship between increased age and sarcopenia was observed after multivariate adjustment. Age was stratified into three tertiles. A dose-dependent relationship between increasing tertiles of age and sarcopenia was observed after adjustment for model 1 (*P* for trend <0.001). The highest tertile of age showed a significantly higher prevalence of sarcopenia, compared with the reference tertile (adjusted OR, 3.375, (95% CI, 1.833–6.214)). The association remained significant but slightly attenuated after adjustment for model 2 (adjusted OR, 2.616, (95% CI, 1.224–5.593); *P* for trend = 0.012), while it was stronger after adjustment for model 3 (adjusted OR, 4.226, (95% CI, 1.561–11.444); *P* for trend = 0.003). However, no significant differences were observed between the first two tertiles.

### 3.3. Association of baPWV with Age Tertiles

The relationship between baPWV and age was confirmed by generalized linear regression models, as shown in Table 3. A significant trend was shown that the elevation of age tertiles was associated with higher baPWV in all three models (*P* for trend < 0.001).

### 3.4. Association between Increased baPWV and Sarcopenia

Similarly, a dose-dependent relationship was also found between elevated baPWV and sarcopenia (Table 4). With 1 SD increase in baPWV, the prevalence of sarcopenia increased by 1.952 (95% CI, 1.544–2.468) after adjusting for model 1, and it remained significant after adjustment for models 2 and 3. Compared with the normal arterial stiffness group, both the borderline arterial stiffness group and the elevated arterial stiffness group showed higher odds of having sarcopenia in model 1, and the OR (95% CI) was 3.081 (1.452–6.539) and 6.363 (2.949–13.729), respectively. Such associations were remained in model 2 and even stronger in model 3.

### 3.5. baPWV Mediates the Association between Increased Age and Sarcopenia: Mediation Analysis

**Figure 1:**
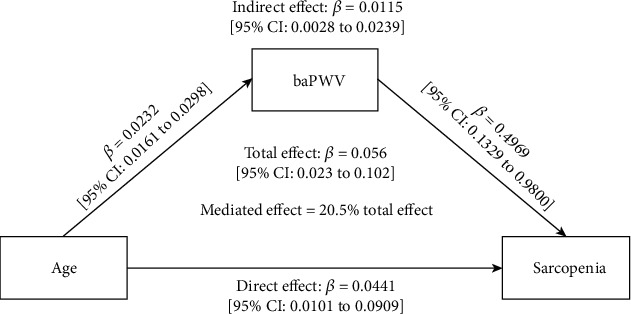
baPWV mediated 20.5% of the positive relationship between increased age and the prevalence of sarcopenia among Chinese patients with T2DM. Adjusted for sex, smoking status (ever or never), drinking status (ever or never), systolic blood pressure, vibration perception threshold classes (<15, ≥15 V), body mass index classes (<24, ≥24 kg/m^2^), alanine transaminase, creatinine, albumin, low-density lipoprotein cholesterol, vitamin D, hemoglobin A1c classes (<7, ≥7%), and diabetes duration classes (≤10, >10 years).

To further explore whether baPWV mediated the association between age and sarcopenia, we conducted the multivariate mediation analysis. After controlling for possible confounding variables, a significant indirect effect was found (*β*, 0.0115, (95% CI, 0.0028–0.0239)), and the direct effect was also significant (*β*, 0.0441, (95% CI, 0.0101–0.0909)), with a significant total effect (*β*, 0.056, (95% CI, 0.023–0.102)). The proportion of the total effect for the association between age and sarcopenia explained by baPWV was 20.5% (Figure 1).

## 4. Discussion

In this cross-sectional hospital-based study, in consistent with former studies, our data demonstrated that as patients age, the prevalence of sarcopenia and arterial stiffness increased, as evaluated by baPWV, among patients with T2DM. Furthermore, a dose-dependent association was found between elevated baPWV and higher prevalence of sarcopenia. By mediation analysis, we observed that age-related increase in the prevalence of sarcopenia was partially mediated by elevated baPWV.

The study was first to investigate the association of age, arterial stiffness, and sarcopenia. One of our findings showed that the prevalence of sarcopenia was higher among older patients, which has been repeatedly reported [2, 3, 23]. Studies showed that age is associated with sarcopenia and its components. Across the life course, muscle mass and grip strength increase in youth but begin to decline in later life [24, 25]. In a Japanese older population, the prevalence of sarcopenia increased significantly with age for both sexes [25, 26]. Our results were consistent with their findings.

Then, our results found that age was associated with higher baPWV, in a dose-dependent manner. Aging is thought to be a dominant determinant of arterial stiffness [27, 28]. As people age, repetitive pulsations result in elastin fiber fatigue and fracture and finally result in arterial dilation and stiffness [29]. The Baltimore Longitudinal Study of Aging collected serial measures of cfPWV among participants aged 21 to 94, rates of cfPWV increase accelerated with advancing age in both sexes [30]. Data from our study confirmed the relationship between increased age and stiffer artery.

Although the age-related presence of sarcopenia and age-related arterial stiffness have been well-documented, evidence on the association between arterial stiffness and sarcopenia is relatively inadequate. Tanaka et al. previously reported that relative skeletal muscle mass index had a negative relation with baPWV; however, such association became marginal after adjusting for %trunk fat or visceral fat area in postmenopausal women with T2DM [31]. Recently, a community-based cross-sectional study aimed at investigating the relationship between baPWV and different components of sarcopenia found that increased baPWV was associated with lower appendicular skeletal muscle index and handgrip strength [32]. Another study including 366 healthy adults in UK has also found a clear increase in arterial stiffness, as evaluated by the cardio-ankle vascular index, associated with increased age and skeletal mass index (SMI) level [33], without accounting for confounding variables, for example, malnutrition, activity, metabolic disorders, and liver and kidney disorders. However, most of the existing studies talked about the association of arterial stiffness and one of the components of sarcopenia. Our study used AWGS 2019 consensus [16] as diagnosis criteria of sarcopenia and determined muscle mass, handgrip strength, and 6-meter walk speed at the same time. Another cross-sectional study performed among community-dwelling participants exploring the relationship of arterial stiffness and sarcopenia using the AWGS 2014 criteria has also demonstrated that increased baPWV was associated with decreased handgrip and lower relative skeletal muscle mass index, as well as higher odds of having sarcopenia but not with 4-meter velocity [34]. Nevertheless, the causal relationship among age, arterial stiffness, and sarcopenia has still not been studied.

By clarifying the role of arterial stiffness in age and sarcopenia using the mediation analysis, the present study tried to add to the already existing evidence. After adjustment for covariates, we observed that baPWV mediated a significant proportion (20.5%) of the positive relationship between increased age and the prevalence of sarcopenia. For the first time, the relationship of arterial stiffness and sarcopenia was examined among T2DM patients. And the present study reported for the first time that arterial stiffness played an intermediating role in the development of age-related sarcopenia among patients with T2DM, suggesting that therapeutic strategies toward arterial stiffness might provide a direction to better prevent sarcopenia and its complications.

Sarcopenia is a newly recognized complication of diabetes, affecting their physical and psychosocial health and reducing their quality of life, especially among older people. Thus, how to prevent the presence and progression of sarcopenia among T2DM requires more attention. Patients with longer diabetes duration were more likely to develop sarcopenia, which was consistent with former literatures [35–37] and might be explained by the long-term effect of hyperglycemia on skeletal muscle [38]. Other factors associated with sarcopenia in individuals with diabetes include age, gender, low BMI, glycemic control, microvascular complications, diabetic foot disease, poor nutritional status, less physical activity, and the use of glucose-lowering drugs [39]. Thus, implementation of energy intake and regular physical exercise is recommended to reduce the risk of sarcopenia among T2DM [39].

Regarding the mediating function of arterial stiffness, there was still no effective method recommended to reduce the frequency of sarcopenia by improving arterial stiffness. We recommend screening for sarcopenia among patients with elevated baPWV or existing arterial stiffness, in patients with T2DM. We also recommend an early practice of healthy vascular aging strategies [40]. Novel strategies including healthy lifestyle strategy and pharmacological agents, such as aerobic exercise, healthy diet pattern, and use of antihypertensive agents and statins, are beneficial for the prevention and treatment of arterial stiffness, and thus, they may also prevent sarcopenia [41].

The mechanism by which age-related arterial stiffness increases sarcopenia among patients with T2DM remains obscure. Inflammation might explain the link of arterial stiffness and sarcopenia. Hyperglycemia and aging-induced inflammation increase arterial stiffness, which then induces more release of inflammatory substances. High level of inflammatory mediators can impair muscle cell maintenance and muscle breakdown [7]. Another possible mechanism may be associated with microvascular dysfunction. During the aging process, arteries become stiffer. Large arterial stiffness then has an intense interaction with microvasculature, resulting in ischemia and microvascular dysfunction [42], where consequently reduced skeletal muscle microvascular function [43], oxidative capacity, and nutrient supply may result in sarcopenia [44]. Further studies on mechanisms of age-related arterial stiffness and sarcopenia are required. These age-related inflammations and arterial stiffness-induced microvascular dysfunctions could be promoted or facilitated by diabetes.

Our current study still had several limitations. First, the current study was a cross-sectional study with a small sample from a single center and confined to Chinese patients with T2DM, which limited the generalizability of the findings. The findings need to be further confirmed by longitudinal studies. Second, we applied baPWV instead of carotid-femoral pulse wave velocity (cfPWV), which is recognized as a gold standard indicator of arterial stiffness [45]. However, baPWV has already been widely used and has been recommended by the American Heart Association [46]. Furthermore, the baPWV assessment was based on a single measurement, which did not allow us to test the reproducibility.

## 5. Conclusions

In summary, we confirmed the significant association between increased age and higher prevalence of sarcopenia among Chinese patients with T2DM. Such association was partially mediated by elevated baPWV. Our study highlights the mediating role of baPWV in the association between aging and sarcopenia, which might provide a new direction for early prevention and probable treatment of sarcopenia in patients with T2DM.

## Figures and Tables

**Table 1:** Characteristics of patients with or without sarcopenia.

Characteristics	Nonsarcopenia (*n* = 357)	Sarcopenia (*n* = 90)	*P* value
SMI (kg/m^2^)	7.60 (6.62-8.30)	6.25 (5.28-6.77)	< 0.001^∗^
Grip strength (kg)	30.2 (22.3-37.9)	21.3 (15.9-26.5)	< 0.001^∗^
Gait speed (m/s)	1.05 (0.92-1.16)	0.83 (0.72-0.96)	< 0.001^∗^
SARC-CalF	1 (0-10)	11 (10-11)	< 0.001^∗^
Male (%)	226 (63.3)	66 (73.3)	0.074
Age (years)	57.0 (48.0-67.0)	65.5 (54.8-74.3)	< 0.001^∗^
Age classes (%)			< 0.001^∗^
≤53 years	129 (36.1)	20 (22.2)	
54–65 years	128 (35.9)	25 (27.8)	
≥66 years	100 (28.0)	45 (50.0)	
BMI (kg/m^2^)	23.9 (22.0-25.9)	21.0 (19.3-23.0)	< 0.001^∗^
BMI classes (%)			< 0.001^∗^
Low (<24 kg/m^2^)	183 (51.3)	75 (83.3)	
High (≥24 kg/m^2^)	174 (48.7)	15 (16.7)	
SBP (mmHg)	132 (120-148)	136 (122-152)	0.407
ALT (U/L)	22 (15-33)	17 (12-30)	0.012^∗^
Creatinine (*μ*mol/L)	65.0 (54.3-81.0)	68.0 (51.5-94.5)	0.332
Albumin (g/L)	38.5 (36.5-40.9)	35.5 (30.8-38.6)	< 0.001^∗^
LDL-c (mmol/L)	2.47 (1.87-3.14)	2.56 (1.86-3.57)	0.247
Vitamin D (nmol/L)	57.9 (45.4-71.1)	54.5 (34.0-65.3)	0.005^∗^
FBG (mmol/L)	8.1 (6.7-9.9)	9.6 (7.5-12.0)	< 0.001^∗^
2hPG (mmol/L)	19.3 ± 4.56	21.1 ± 4.87	0.003^∗^
HbA1c (%)	9.2 (7.7-11.2)	9.1 (8.2-11.5)	0.575
HbA1c classes (%)			0.732
<7%	40 (11.3)	9 (10.0)	
≥7%	315 (88.7)	81 (90.0)	
VPT classes (%)			< 0.001^∗^
Low (<15 V)	262 (79.6)	43 (53.8)	
High (≥15 V)	67 (20.4)	37 (46.3)	
baPWV (cm/s)	1557 (1355-1810)	1766 (1548-2132)	< 0.001^∗^
baPWV classes (%)			< 0.001^∗^
Normal (<1400 cm/s)	112 (31.4)	10 (11.1)	
Boardline (1400 to <1800 cm/s)	154 (43.1)	38 (42.2)	
Elevated (≥1800 cm/s)	91 (25.5)	42 (46.7)	
Diabetes duration (years)	9.0 (3.0-14.0)	10.0 (5.0-20.0)	0.001^∗^
Diabetes duration classes (%)			0.001^∗^
≤10 years	251 (70.3)	47 (52.2)	
>10 years	106 (29.7)	43 (47.8)	
Smoking status (%)			0.088
Never	214 (59.9)	45 (50.0)	
Ever	143 (40.1)	45 (50.0)	
Drinking status (%)			0.436
Never	210 (58.8)	57 (63.3)	
Ever	147 (41.2)	33 (36.7)	

SMI: skeletal mass index; SBP: systolic blood pressure; ALT: alanine transaminase; LDL-c: low density lipoprotein-cholesterol; FBG: fasting blood glucose; 2hPG: 2-hour postload plasma glucose; HbA1c: hemoglobin A1c; BMI: body mass index; VPT: vibration perception threshold; baPWV: brachial-ankle pulse wave velocity.

**Table 2:** Multivariate logistic regression models for increased ages and higher prevalence of sarcopenia.

	Sarcopenia/nonsarcopenia	Adjusted ORs (95% CI)		
		Model 1	Model 2	Model 3
Age (years)	90/357	1.049 (1.028-1.070)^∗^	1.040 (1.1014-1.066)^∗^	1.057 (1.024-1.091)^∗^
Age classes (years)				
≤53	20/129	Reference	Reference	Reference
54–65	25/128	1.364 (0.713-2.610)	1.218 (0.596-2.488)	1.460 (0.602-3.536)
≥66	45/100	3.375 (1.833-6.214)^∗^	2.616 (1.224-5.593)^∗^	4.226 (1.561-11.444)^∗^
*P* for trend	NA	< 0.001^∗^	0.012^∗^	0.003^∗^

Model 1: adjusted for sex, smoking status (ever or never), and drinking status (ever or never). Model 2: model 1 plus adjustment for systolic blood pressure and vibration perception threshold classes (<15, ≥15 V). Model 3: model 2 plus adjustment for body mass index classes (<24, ≥24 kg/m^2^), alanine transaminase, creatinine, albumin, low-density lipoprotein cholesterol, vitamin D, hemoglobin A1c classes (<7, ≥7%), and diabetes duration classes (≤10, >10 years).

**Table 3:** Multivariable-adjusted differences of baPWV values by three tertiles of age.

baPWV+1 SD	Age classes (years)			*P* for trend
	≤53	54–65	≥66	
Model 1	Reference	1.572 (1.282-1.928)^∗^	3.764 (3.032-4.642)^∗^	< 0.001^∗^
Model 2	Reference	1.365 (1.125-1.657)^∗^	2.616 (2.074-3.299)^∗^	< 0.001^∗^
Model 3	Reference	1.203 (0.993-1.458)	2.137 (1.685-2.710)^∗^	< 0.001^∗^

Model 1: adjusted for sex, smoking status (ever or never), and drinking status (ever or never). Model 2: model 1 plus adjustment for systolic blood pressure and vibration perception threshold classes (<15, ≥15 V). Model 3: model 2 plus adjustment for body mass index classes (<24, ≥24 kg/m^2^), alanine transaminase, creatinine, albumin, low-density lipoprotein cholesterol, vitamin D, hemoglobin A1c classes (<7, ≥7%), and diabetes duration classes (≤10, >10 years).

**Table 4:** Multivariate logistic regression models for elevated baPWV and higher prevalence of sarcopenia.

	Sarcopenia/nonsarcopenia	Adjusted ORs (95% CI)		
		Model 1	Model 2	Model 3
baPWV+1 SD	90/357	1.952 (1.544-2.468)^∗^	1.750 (1.316-2.325)^∗^	1.878 (1.308-2.695)^∗^
baPWV classes (cm/s)				
Normal (<1400)	10/112	Reference	Reference	Reference
Boardline (1400 to <1800)	38/154	3.081 (1.452-6.539)^∗^	3.444 (1.491-7.952)^∗^	5.077 (1.809-14.244)^∗^
Elevated (≥1800)	42/91	6.363 (2.949-13.729)^∗^	5.103 (2.037-12.785)^∗^	7.728 (2.466-24.221)^∗^
*P* for trend	NA	< 0.001^∗^	0.001^∗^	0.001^∗^

Model 1: adjusted for sex, smoking status (ever or never), and drinking status (ever or never). Model 2: model 1 plus adjustment for systolic blood pressure and vibration perception threshold classes (<15, ≥15 V). Model 3: model 2 plus adjustment for body mass index classes (<24, ≥24 kg/m^2^), alanine transaminase, creatinine, albumin, low-density lipoprotein cholesterol, vitamin D, hemoglobin A1c classes (<7, ≥7%), and diabetes duration classes (≤10, >10 years).

## Data Availability

Data that support the findings of this study are available from Hong Zhu upon reasonable request and approval.

## Abbreviations

T2DM: Type 2 diabetes mellitus
baPWV: Brachial-ankle pulse wave velocity
PWV: Pulse wave velocity
cfPWV: Carotid-femoral pulse wave velocity
ABI: Ankle-brachial index
eGFR: Estimated glomerular filtration rate
AWGS: Asian Working Group for Sarcopenia
BMI: Body mass index
SBP: Systolic blood pressure
ALT: Alanine transaminase
LDL-c: Low-density lipoprotein cholesterol
HbA1c: Hemoglobin A1c
FBG: Fasting blood glucose
2hPG: Two-hour postload plasma glucose
VPT: Vibration perception threshold
OR: Odds ratio
CI: Confidence interval
SMI: Skeletal mass index.

## References

[1] Rosenberg I. H. (1997). Sarcopenia: origins and clinical relevance. *The Journal of Nutrition*.

[2] Gielen E., O'Neill T. W., Pye S. R. (2015). Endocrine determinants of incident sarcopenia in middle-aged and elderly European men. *Journal of Cachexia, Sarcopenia and Muscle*.

[3] Ferrucci L., de Cabo R., Knuth N. D., Studenski S. (2012). Of Greek heroes, wiggling worms, mighty mice, and old body builders. *The Journals of Gerontology. Series A, Biological Sciences and Medical Sciences*.

[4] Schellinger I. N., Mattern K., Raaz U. (2019). The hardest part. *Arteriosclerosis, Thrombosis, and Vascular Biology*.

[5] Lu Y., Pechlaner R., Cai J. (2020). Trajectories of age-related arterial stiffness in Chinese men and women. *Journal of the American College of Cardiology*.

[6] Wu S., Jin C., Li S. (2019). Aging, arterial stiffness, and blood pressure association in Chinese adults. *Hypertension*.

[7] Tap L., Kirkham F. A., Mattace-Raso F., Joly L., Rajkumar C., Benetos A. (2020). Unraveling the links underlying arterial stiffness, bone demineralization, and muscle loss. *Hypertension*.

[8] Kohara K., Okada Y., Ochi M. (2017). Muscle mass decline, arterial stiffness, white matter hyperintensity, and cognitive impairment: Japan Shimanami health promoting program study. *Journal of Cachexia, Sarcopenia and Muscle*.

[9] Anagnostis P., Gkekas N. K., Achilla C. (2020). Type 2 diabetes mellitus is associated with increased risk of sarcopenia: a systematic review and meta-analysis. *Calcified Tissue International*.

[10] Schram M. T., Henry R. M. A., van Dijk R. A. J. M. (2004). Increased central artery stiffness in impaired glucose metabolism and type 2 diabetes: the Hoorn study. *Hypertension*.

[11] Cruickshank K., Riste L., Anderson S. G., Wright J. S., Dunn G., Gosling R. G. (2002). Aortic pulse-wave velocity and its relationship to mortality in diabetes and glucose intolerance: an integrated index of vascular function?. *Circulation*.

[12] Chamberlain J. J., Rhinehart A. S., Shaefer C. F., Neuman A. (2016). Diagnosis and management of diabetes: synopsis of the 2016 American Diabetes Association Standards of Medical Care in Diabetes. *Annals of Internal Medicine*.

[13] Munakata M. (2016). Brachial-ankle pulse wave velocity: background, method, and clinical evidence. *Pulse (Basel)*.

[14] Fu X., Tian Z., Thapa S. (2020). Comparing SARC-F with SARC-CalF for screening sarcopenia in advanced cancer patients. *Clinical Nutrition*.

[15] Lim W. S., Chew J., Lim J. P., Tay L., Hafizah N., Ding Y. Y. (2019). Letter to the editor: case for validated instead of standard cut-offs for SARC-CalF. *The Journal of Nutrition, Health & Aging*.

[16] Chen L.-K., Woo J., Assantachai P. (2020). Asian Working Group for Sarcopenia: 2019 consensus update on sarcopenia diagnosis and treatment. *Journal of the American Medical Directors Association*.

[17] Liu Y., Lai X., Guo W. (2020). Total white blood cell count mediated the association between increased arterial stiffness and risk of type 2 diabetes mellitus in Chinese adults. *Arteriosclerosis, Thrombosis, and Vascular Biology*.

[18] Takashima N., Turin T. C., Matsui K. (2014). The relationship of brachial-ankle pulse wave velocity to future cardiovascular disease events in the general Japanese population: the Takashima study. *Journal of Human Hypertension*.

[19] Zheng M., Zhang X., Chen S. (2020). Arterial stiffness preceding diabetes: a longitudinal study. *Circulation Research*.

[20] Dong Z., Tan Z., Chen Z. (2021). Association of BMI and lipid profiles with axillary osmidrosis: a retrospective case-control study. *The Journal of Dermatological Treatment*.

[21] Garrow A. P., Boulton A. J. M. (2006). Vibration perception threshold--a valuable assessment of neural dysfunction in people with diabetes. *Diabetes/Metabolism Research and Reviews*.

[22] Koyanagi A., Veronese N., Solmi M. (2020). Fruit and vegetable consumption and sarcopenia among older adults in low- and middle-income countries. *Nutrients*.

[23] Zhang Y., Chen X., Hou L. (2020). Prevalence and risk factors governing the loss of muscle function in elderly sarcopenia patients: a longitudinal study in China with 4 years of follow-up. *The Journal of Nutrition, Health & Aging*.

[24] Dennison E. M., Sayer A. A., Cooper C. (2017). Epidemiology of sarcopenia and insight into possible therapeutic targets. *Nature Reviews Rheumatology*.

[25] Shaw S. C., Dennison E. M., Cooper C. (2017). Epidemiology of sarcopenia: determinants throughout the lifecourse. *Calcified Tissue International*.

[26] Nakamura K., Yoshida D., Honda T. (2021). Prevalence and mortality of sarcopenia in a community-dwelling older Japanese population: the Hisayama study. *Journal of Epidemiology*.

[27] McEniery C. M., Yasmin H. I. R., Qasem A., Wilkinson I. B., Cockcroft J. R. (2005). Normal vascular aging: differential effects on wave reflection and aortic pulse wave velocity: the Anglo-Cardiff Collaborative Trial (ACCT). *Journal of the American College of Cardiology*.

[28] Wilkinson I. B., McEniery C. M. (2012). Arteriosclerosis. *Hypertension*.

[29] O'Rourke M. F., Hashimoto J. (2007). Mechanical factors in arterial aging: a clinical perspective. *Journal of the American College of Cardiology*.

[30] AlGhatrif M., Strait J. B., Morrell C. H. (2013). Longitudinal trajectories of arterial stiffness and the role of blood pressure: the Baltimore Longitudinal Study of Aging. *Hypertension*.

[31] Tanaka K.-I., Kanazawa I., Sugimoto T. (2016). Reduced muscle mass and accumulation of visceral fat are independently associated with increased arterial stiffness in postmenopausal women with type 2 diabetes mellitus. *Diabetes Research and Clinical Practice*.

[32] Rong Y.-D., Bian A.-L., Hu H.-Y., Ma Y., Zhou X.-Z. (2020). A cross-sectional study of the relationships between different components of sarcopenia and brachial ankle pulse wave velocity in community-dwelling elderly. *BMC Geriatrics*.

[33] Kirkham F. A., Bunting E., Fantin F., Zamboni M., Rajkumar C. (2019). Independent association between cardio-ankle vascular index and sarcopenia in older U.K. adults. *Journal of the American Geriatrics Society*.

[34] Zhang L., Guo Q., Feng B. L. (2019). A cross-sectional study of the association between arterial stiffness and sarcopenia in Chinese community-dwelling elderly using the Asian working group for sarcopenia criteria. *The Journal of Nutrition, Health & Aging*.

[35] Sazlina S. G., Lee P. Y., Chan Y. M., Hamid M. S. A., Tan N. C. (2020). The prevalence and factors associated with sarcopenia among community living elderly with type 2 diabetes mellitus in primary care clinics in Malaysia. *PLoS One*.

[36] Kaji A., Hashimoto Y., Kobayashi Y. (2019). Sarcopenia is associated with tongue pressure in older patients with type 2 diabetes: a cross-sectional study of the KAMOGAWA-DM cohort study. *Geriatrics & Gerontology International*.

[37] Qiao Y.-S., Chai Y.-H., Gong H.-J. (2021). The association between diabetes mellitus and risk of sarcopenia: accumulated evidences from observational studies. *Front Endocrinol (Lausanne)*.

[38] Volpato S., Bianchi L., Lauretani F. (2012). Role of muscle mass and muscle quality in the association between diabetes and gait speed. *Diabetes Care*.

[39] Izzo A., Massimino E., Riccardi G., Della P. G. (2021). A narrative review on sarcopenia in type 2 diabetes mellitus: prevalence and associated factors. *Nutrients*.

[40] Currie G., Delles C. (2017). Healthy vascular aging. *Hypertension*.

[41] Nowak K. L., Rossman M. J., Chonchol M., Seals D. R. (2018). Strategies for achieving healthy vascular aging. *Hypertension*.

[42] Rensma S. P., Stehouwer C. D. A., Van Boxtel M. P. J. (2020). Associations of arterial stiffness with cognitive performance, and the role of microvascular dysfunction: the Maastricht study. *Hypertension*.

[43] Cooper L. L., Musani S. K., Washington F. (2018). Relations of microvascular function, cardiovascular disease risk factors, and aortic stiffness in blacks: the Jackson Heart study. *Journal of the American Heart Association*.

[44] Prior S. J., Ryan A. S., Blumenthal J. B., Watson J. M., Katzel L. I., Goldberg A. P. (2016). Sarcopenia is associated with lower skeletal muscle capillarization and exercise capacity in older adults. *The Journals of Gerontology. Series A, Biological Sciences and Medical Sciences*.

[45] Erbel R., Aboyans V., Boileau C. (2014). 2014 ESC guidelines on the diagnosis and treatment of aortic diseases: document covering acute and chronic aortic diseases of the thoracic and abdominal aorta of the adult. The task force for the diagnosis and treatment of aortic diseases of the European Society of Cardiology (ESC). *European Heart Journal*.

[46] Townsend R. R., Wilkinson I. B., Schiffrin E. L. (2015). Recommendations for improving and standardizing vascular research on arterial stiffness: a scientific statement from the American Heart Association. *Hypertension*.

